# Supplementary material for: Systemic Anti-Cancer Therapy in Synovial Sarcoma: A Systematic Review
Source: Cancers (Basel). 2018 Nov 1;10(11):417. doi: 10.3390/cancers10110417 (PMC6267101; doi:10.3390/cancers10110417)
Supplement: Supplementary file 1 [file cancers-10-00417-s001.pdf]

cancers

Supplementary material

Systemic Anti-Cancer Therapy in Synovial Sarcoma: A Systematic Review

Richard F. Riedel, Robin L. Jones, Antoine Italiano, Chet Bohac, Juliette C. Thompson, Kerstin Mueller, Zaeem Khan, Seth M. Pollack and Brian A. Van Tine

Table S1. Search Strategies implemented in each database. Note that the search was originally designed to also include myxoid and round cell liposarcoma.

Database searched: Ovid MEDLINE(R) Epub Ahead of Print, In-Process & Other Non-Indexed Citations, Ovid MEDLINE(R) Daily, Ovid MEDLINE and Versions(R) [Searched 19th April 2017; updated 30th January 2018]		
#	**Search terms**	**Results returned**
1	Sarcoma, Synovial/	2958
2	((synovi* or tendosynovial) adj2 (sarcoma or malignant)).tw.	3175
3	(synoviasarcoma or synoviosarcoma or synovioma).tw.	211
4	Liposarcoma/	3965
5	(myxosarcoma or myxoid liposarcoma or round cell liposarcoma or MRCLS).tw.	966
6	mixed type liposarcoma.tw.	28
7	or/1-6	8587
8	Case study/ or case reports/	1,878,316
9	(case adj2 (stud * or report *)).tw.	588,258
10	8 or 9	2,141,590
11	7 not 10	4602
12	limit 11 to yr="2000-current" [April 19 2017] *	2353
Database searched: Embase 1974 to 2017 [Searched 19th April 2017; updated 30th January 2018]		
#	**Search terms**	**Results returned**
1	exp * synovial sarcoma/	2325
2	((synovi* or tendosynovial) adj2 (sarcoma or malignant)).tw.	3953
3	(synoviasarcoma or synoviosarcoma or synovioma).tw.	193
4	exp * Liposarcoma/	3934
5	(myxosarcoma or myxoid liposarcoma or round cell liposarcoma or MRCLS).tw.	1177
6	mixed type liposarcoma.tw.	36
7	or/1-6	8957
8	Case study/ or case report/	2,307,885
9	(case adj2 (stud * or report *)).tw.	750,563
10	8 or 9	2,526,844
11	7 not 10	4737
12	limit 11 to yr="2000 -Current" [April 19 2017] *	3116
13	conference.so.	2,542,009
14	12 not 13	2225
Database searched: EBM Reviews - Cochrane Central Register of Controlled Trials [Searched 19th April 2017; updated 30th January 2018]		
#	**Search terms**	**Results returned**
1	sarcoma, synovial/	0
2	((synovi* or tendosynovial) adj2 (sarcoma or malignant)).tw.	22
3	(synoviasarcoma or synoviosarcoma or synovioma).tw.	0

4	Liposarcoma/	6
5	(myxosarcoma or myxoid liposarcoma or round cell liposarcoma or MRCLS).tw.	5
6	mixed type liposarcoma.tw.	1
7	or/1-6 [April 19 2017] *	32

* Searches were updated on 30th January 2018; the following numbers of additional references were identified from all databases combined (From 1st January 2017-30th January 2018): 408 abstracts.

Table S2. Reported objectives of studies included in review.

Author, Publication Year	Objectives
**Localized**	
De Silva, 2004 [1]	To study the association of clinicopathologic variables with recurrence, metastases, and tumor-related death in patients with synovial sarcoma who did not have metastases at presentation.
Scheer, 2016 [2]	Assess the outcomes, identify prognostic factors, and to analyse treatment strategies in synovial sarcoma patients with metastases at diagnosis.
Krieg, 2011 [3]	To investigate the extent to which individual clinical tumor-specific factors as well as surgical approach affect the outcome of patients with synovial sarcoma with at least 10-year follow-up.
Beaino, 2016 [4]	To determine the local recurrence-free survival and distant recurrence-free survival in patients with T1 (<5 cm) localized synovial sarcoma; To identify what determines local recurrence and metastasis; To assess if radiation and chemotherapy affect local recurrence and metastasis in early stage disease.
Orbach, 2011 [5]	To determine if the modified indications for radiotherapy or radical surgery, according to the quality of initial resection and response to initial chemotherapy, had an impact on survival.
Shi, 2013 [6]	To review the outcomes of a cohort of synovial sarcoma patients at single institution, identify prognostic factors impacting both local and distant disease control, and document acute and late toxicity related to treatment.
Eilber, 2007 [7]	To determine if ifosfamide-based chemotherapy offers a survival benefit to adult patients with primary extremity synovial sarcoma.
Al-Hussaini, 2011 [8]	To investigate the impact of chemotherapy on survival in both paediatric and adult patients with localized synovial sarcoma treated at two specialized sarcoma centres.
Trassard, 2001 [9]	To identify most significant and therapeutically relevant prognostic factors in adults with localized primary synovial sarcomas and to confirm the usefulness of the French Federation of Cancer Centers (FNCLCC) grading system.
Ferrari, 2015 [10]	To assess survival rates and treatment failure patterns; the role of ifosfamide–doxorubicin chemotherapy in improving response rates in patients with unresectable synovial sarcoma; and to determine the impact of omitting adjuvant chemotherapy in patients with low-risk synovial sarcoma.
Brecht, 2006 [11]	To identify risk and treatment factors which influence survival rates in patients with synovial sarcoma.
Italiano, 2009 [12]	To clarify the prognosis factors and the impact of neo-adjuvant/adjuvant chemotherapy for adult patients with localized synovial sarcoma.
Canter, 2008 [13]	To analyse the clinicopathologic predictors of distant recurrence and sarcoma-specific death.
Vlenterie, 2015 [14]	To explore age as a prognostic factor in synovial sarcoma patients.
Vining, 2017 [15]	To study the effect of adjuvant chemotherapy on OS among a large cohort of patients undergoing curative-intent resection of synovial sarcoma.
Gronchi, 2017 [16]	Show the superiority of the neoadjuvant administration of histotype-tailored regimen to standard chemotherapy.
**Locally advanced or metastatic**	

Takenaka, 2008 [17]	To clarify the prognostic impact of SYTY-SSX fusion type, in association with other clinical factors, in patients with synovial sarcoma in Japan.
Setsu, 2013 [18]	To investigate the phosphorylation status of Akt (protein kinase B), mTOR, 4E-BP1, and S6 in a large series of synovial sarcoma and evaluated the relation between Akt/mTOR pathway activation and clinical and histopathologic features.
Deshmukh, 2004 [19]	To determine whether distal or truncal location of synovial sarcoma is of prognostic significance for survival when corrected for tumor size.
Guillou, 2004 [20]	To assess the prognostic value of SYT-SSX fusion type, in comparison with other factors, in patients with synovial sarcoma.
Palmerini, 2009 [21]	To retrospectively examine all synovial sarcoma patients treated at our institution to identify tumor-related and treatment-related factors influencing survival.
Ferrari, 2004 [22]	To retrospectively analyse a large series of synovial sarcoma patients of all ages who were treated at a single centre.
Vlenterie, 2016 [23]	Compared the demographics and outcome of a large subset of advanced synovial sarcoma patients treated in first-line palliative chemotherapy studies between 1976 and 2012 and compared these results with other advanced STS patients treated in these studies.
Brennan, 2016 [24]	To assess the role of age, socioeconomic status and other prognostic factors on outcome for synovial sarcoma.
Corey, 2014 [25]	To determine demographic and survivorship of 34 soft tissue sarcomas.
Spurrell, 2005 [26]	To look specifically at a cohort of patients with advanced synovial sarcoma and to identify potential prognostic factors.
**Metastatic disease**	
Sanfilippo, 2015 [27]	To review all patients with advanced synovial sarcoma treated with trabectedin at four European sarcoma reference centers and within the Italian Rare Cancer Network.
Savina, 2017 [28]	To utilized a unique set of data to assess the modalities of treatment of patients with metastatic STS in a real-life setting, to evaluate their impact on the outcome according to the histological subtype.

References

1. De Silva, M.V.C.; McMahon, A.D.; Reid, R. Prognostic Factors Associated with Local Recurrence, Metastases, and Tumor-Related Death in Patients with Synovial Sarcoma. *Am. J. Clin. Oncol.* **2004**, *27*, 113–121.
2. Scheer, M.; Dantonello, T.; Hallmen, E.; Blank, B.; Sparber-Sauer, M.; Vokuhl, C.; Leuschner, I.; Munter, M.W.; von Kalle, T.; Bielack, S.S.; et al. Synovial Sarcoma Recurrence in Children and Young Adults. *Ann. Surg. Oncol.* **2016**, *23*, 618–626.
3. Krieg, A.H.; Hefti, F.; Speth, B.M.; Jundt, G.; Guillou, L.; Exner, U.G.; von Hochstetter, A.R.; Cserhati, M.D.; Fuchs, B.; Mouhsine, E.; et al. Synovial sarcomas usually metastasize after >5 years: A multicenter retrospective analysis with minimum follow-up of 10 years for survivors. *Ann. Oncol.* **2011**, *22*, 458–467.
4. Beaino, M.E.; Araujo, D.M.; Gopalakrishnan, V.; Lazar, A.J.; Lin, P.P. Prognosis of T1 synovial sarcoma depends upon surgery by oncologic surgeons. *J.Surg. Oncol.* **2016**, *114*, 490–494.
5. Orbach, D.; McDowell, H.; Rey, A.; Bouvet, N.; Kelsey, A.; Stevens, M.C. Sparing strategy does not compromise prognosis in pediatric localized synovial sarcoma: Experience of the International Society of pediatric oncology, malignant mesenchymal tumors (SIOP-MMT) Working Group. *Pediatr. Blood Cancer* **2011**, *57*, 1130–1136.
6. Shi, W.; Indelicato, D.J.; Morris, C.G.; Scarborough, M.T.; Gibbs, C.P.; Zlotecki, R.A. Long-term treatment outcomes for patients with synovial sarcoma: A 40-year experience at the university of Florida. *Am. J. Clin. Oncol.* **2013**, *36*, 83–88.
7. Eilber, F.C.; Brennan, M.F.; Eilber, F.R.; Eckardt, J.J.; Grobmyer, S.R.; Riedel, E.; Forscher, C.; Maki, R.G.; Singer, S. Chemotherapy is associated with improved survival in adult patients with primary extremity synovial sarcoma. *Ann. Surg.* **2007**, *246*, 105–113.
8. Al-Hussaini, H.; Hogg, D.; Blackstein, M.E.; O'Sullivan, B.; Catton, C.N.; Chung, P.W.; Griffin, A.M.; Hodgson, D.; Hopyan, S.; Kandel, R.; et al. Clinical features, treatment, and outcome in 102 adult and pediatric patients with localized high-grade synovial sarcoma. *Sarcoma* **2011**, *2011*, 231789.

9. Trassard, M.; Le Doussal, V.; Hacene, K.; Terrier, P.; Ranchere, D.; Guillou, L.; Fiche, M.; Collin, F.; Vilain, M.O.; Bertrand, G.; et al. Prognostic factors in localized primary synovial sarcoma: A multicenter study of 128 adult patients. *J. Clin. Oncol.* **2001**, *19*, 525–534.
10. Ferrari, A.; de Salvo, G.L.; Brennan, B.; van Noesel, M.M.; De Paoli, A.; Casanova, M.; Francotte, N.; Kelsey, A.; Alaggio, R.; Oberlin, O.; et al. Synovial sarcoma in children and adolescents: The European Pediatric Soft Tissue Sarcoma Study Group prospective trial (EpSSG NRSTS 2005). *Ann. Oncol.* **2015**, *26*, 567–572.
11. Brecht, I.B.; Ferrari, A.; Int-Veen, C.; Schuck, A.; Mattke, A.C.; Casanova, M.; Bisogno, G.; Carli, M.; Koscielniak, E.; Treuner, J. Grossly-resected synovial sarcoma treated by the German and Italian pediatric soft tissue sarcoma cooperative groups: Discussion on the role of adjuvant therapies. *Pediatr. Blood Cancer* **2006**, *46*, 11–17.
12. Italiano, A.; Penel, N.; Robin, Y.M.; Bui, B.; Le Cesne, A.; Piperno-Neumann, S.; Tubiana-Hulin, M.; Bompas, E.; Chevreau, C.; Isambert, N.; et al. Neo/adjuvant chemotherapy does not improve outcome in resected primary synovial sarcoma: A study of the French Sarcoma Group. *Ann. Oncol.* **2009**, *20*, 425–430.
13. Canter, R.J.; Qin, L.X.; Maki, R.G.; Brennan, M.F.; Ladanyi, M.; Singer, S. A synovial sarcoma-specific preoperative nomogram supports a survival benefit to ifosfamide-based chemotherapy and improves risk stratification for patients. *Clin. Cancer Res.* **2008**, *14*, 8191–8197.
14. Vlenterie, M.; Ho, V.K.Y.; Kaal, S.E.J.; Vlenterie, R.; Haas, R.; Van Der Graaf, W.T.A. Age as an independent prognostic factor for survival of localised synovial sarcoma patients. *Br. J. Cancer* **2015**, *113*, 1602–1606.
15. Vining, C.C.; Sinnamon, A.J.; Ecker, B.L.; Kelz, R.R.; Fraker, D.L.; Roses, R.E.; Karakousis, G.C., Adjuvant chemotherapy in resectable synovial sarcoma. *J. Surg. Oncol.* **2017**, *116*, 550–558.
16. Gronchi, A.; Ferrari, S.; Quagliuolo, V.; Broto, J.M.; Pousa, A.L.; Grignani, G.; Basso, U.; Blay, J.Y.; Tendero, O.; Beveridge, R.D.; et al. Histotype-tailored neoadjuvant chemotherapy versus standard chemotherapy in patients with high-risk soft-tissue sarcomas (ISG-STS 1001): An international, open-label, randomised, controlled, phase 3, multicentre trial. *Lancet Oncol.* **2017**, *18*, 812–822.
17. Takenaka, S.; Ueda, T.; Naka, N.; Araki, N.; Hashimoto, N.; Myoui, A.; Ozaki, T.; Nakayama, T.; Toguchida, J.; Tanaka, K.; et al. Prognostic implication of SYT-SSX fusion type in synovial sarcoma: A multi-institutional retrospective analysis in Japan. *Oncol. Rep.* **2008**, *19*, 467–476.
18. Setsu, N.; Kohashi, K.; Fushimi, F.; Endo, M.; Yamamoto, H.; Takahashi, Y.; Yamada, Y.; Ishii, T.; Yokoyama, K.; Iwamoto, Y.; et al Prognostic impact of the activation status of the Akt/mTOR pathway in synovial sarcoma. *Cancer* **2013**, *119*, 3504–3513.
19. Deshmukh, R.; Mankin, H.J.; Singer, S. Synovial Sarcoma: The Importance of Size and Location for Survival. *Clin. Orthop. Relat. Res.* **2004**, *419*, 155–161.
20. Guillou, L.; Benhattar, J.; Bonichon, F.; Gallagher, G.; Terrier, P.; Stauffer, E.; De Saint Aubain Somerhausen, N.; Michels, J.J.; Jundt, G.; Vince, D.R.; et al. Histologic grade, but not SYT-SSX fusion type, is an important prognostic factor in patients with synovial sarcoma: A multicenter, retrospective analysis. *J. Clin. Oncol.* **2004**, *22*, 4040–4050.
21. Palmerini, E.; Staals, E.L.; Alberghini, M.; Zanella, L.; Ferrari, C.; Benassi, M.S.; Picci, P.; Mercuri, M.; Bacci, G.; Ferrari, S. Synovial sarcoma: Retrospective analysis of 250 patients treated at a single institution. *Cancer* **2009**, *115*, 2988–2998.
22. Ferrari, A.; Gronchi, A.; Casanova, M.; Meazza, C.; Gandola, L.; Collini, P.; Lozza, L.; Bertulli, R.; Olmi, P.; Casali, P.G. Synovial sarcoma: A retrospective analysis of 271 patients of all ages treated at a single institution. *Cancer* **2004**, *101*, 627–634.
23. Vlenterie, M.; Litiere, S.; Rizzo, E.; Marreaud, S.; Judson, I.; Gelderblom, H.; Le Cesne, A.; Wardelmann, E.; Messiou, C.; Gronchi, A.; et al. Outcome of chemotherapy in advanced synovial sarcoma patients: Review of 15 clinical trials from the European Organisation for Research and Treatment of Cancer Soft Tissue and Bone Sarcoma Group; Setting a new landmark for studies in this entity. *Eur. J. Cancer* **2016**, *58*, 62–72.
24. Brennan, B.; Stiller, C.; Grimer, R.; Dennis, N.; Broggio, J.; Francis, M. Outcome and the effect of age and socioeconomic status in 1318 patients with synovial sarcoma in the English National Cancer Registry: 1985–2009. *Clin. Sarcoma Res.* **2016**, *6*, 18.
25. Corey, R.M.; Swett, K.; Ward, W.G. Epidemiology and survivorship of soft tissue sarcomas in adults: A national cancer database report. *Cancer Med.* **2014**, *3*, 1404–1415.
26. Spurrell, E.L.; Fisher, C.; Thomas, J.M.; Judson, I.R. Prognostic factors in advanced synovial sarcoma: An analysis of 104 patients treated at the Royal Marsden Hospital. *Ann. Oncol.* **2005**, *16*, 437–444.
27. Sanfilippo, R.; Dileo, P.; Blay, J.Y.; Constantinidou, A.; Le Cesne, A.; Benson, C.; Vizzini, L.; Contu, M.; Baldi, G.G.; Dei Tos, A.P.; et al. Trabectedin in advanced synovial sarcomas: A multicenter retrospective

study from four European institutions and the Italian Rare Cancer Network. *Anticancer Drugs* **2015**, *26*, 678–681.

28. Savina, M.; Le Cesne, A.; Blay, J.Y.; Ray-Coquard, I.; Mir, O.; Toulmonde, M.; Cousin, S.; Terrier, P.; Ranchere-Vince, D.; Meeus, P.; et al. Patterns of care and outcomes of patients with METAstatic soft tissue SARComa in a real-life setting: The METASARC observational study. *BMC Med.* **2017**, *15*, 78.

© 2018 by the authors. Licensee MDPI, Basel, Switzerland. This article is an open access article distributed under the terms and conditions of the Creative Commons Attribution (CC BY) license (http://creativecommons.org/licenses/by/4.0/).
